# The Influence of One Disease upon Another

**Published:** 1899-07-15

**Authors:** 


### THE INFLUENCE OF ONE DISEASE UPON ANOTHER.

Nothing is more curious than the way in which "health"—if we may presume to personify a condition —keeps at bay, or at least holds in subjection, maladies, the germs of which we carry within us. Of the non-pathogenic microbes which we carry about with us the name perhaps is legion, and even of pathogenic ones there can be little doubt that we often carry a goodly store, which probably accounts for the onset of distinctly microbic maladies when from any cause—typically from prolonged chill—the body resistance is lowered or destroyed. Perhaps it is in this way that we must explain the very curious fact, pointed out by Mr. Warrington Haward,[1] that a recrudescence of a latent or quiescent syphilis is apt to occur after influenza. In this, he says, influenza curiously resembles malarial fever, which has a notable power of bringing out some of the later symptoms of syphilis, and making manifest any remaining syphilitic taint. A case is described in which a woman, 42 years of age, had been infected 18 years before, the primary sore being followed by well-marked secondary symptoms lasting a year and a half. During this time she was under constant treatment. Then for a year she remained free from symptoms, but the following year she again suffered from ulceration of the throat and skin, for which she was treated. After this she remained well, at least she could remember no noticeable symptoms until a recent attack of influenza, during which rupial bullæ appeared over the body and developed into numerous and extensive ulcers, her hair fell off so that she became almost completely bald, and her complexion underwent a notable change from the pallor of anæmia to a dull bistre tint. Then ulceration of the soft palate occurred, leading to perforation, a large periosteal node formed over the back of the radius, and she had ulcers between the toes. Under calomel baths, iodides, tonics, and good diet she made a slow but satisfactory recovery. The matter is one of very great interest, as showing what very complicated things diseases may sometimes be. The "disease" or particular infection may breed true enough; but it may liberate in its course a whole crowd of enemies which had before been imprisoned and held in durance by virtue of our "vitality" or power of resistance, by phagocytes, defensive proteids, or whatever name we choose to use as explaining our capacity for keeping well. Infection from without is not the sole enemy which man has to fear, but rather the mass of insurgents from within his own territory, often, doubtless, the products of old misgovernment, which, so soon as infection invades his tissues, rally to the standard.

Lancet, July 1.

